# Supplementary material for: High-throughput framework for genetic analyses of adverse drug reactions using electronic health records
Source: PLoS Genet. 2021 Jun 1;17(6):e1009593. doi: 10.1371/journal.pgen.1009593 (PMC8195357; doi:10.1371/journal.pgen.1009593)
Supplement: S1 Table — (PDF) [file pgen.1009593.s001.pdf]

S1 Table. Summary of types of adverse drug reactions stratified by self-reported ancestry.

Drug	Rash	Cough	Anaphylaxis	Myalgia	Swelling	Nausea	GI	Asthma	Bleeding	Missing	Total
European Ancestry											
Penicillin	5889 (47.9)	7 (0.1)	673 (5.5)	12 (0.1)	973 (7.9)	539 (4.4)	517 (4.2)	6 (0.0)	15 (0.1)	4573 (37.2)	12294
Sulfa	3491 (41.1)	7 (0.1)	235 (2.8)	33 (0.4)	564 (6.6)	593 (7.0)	226 (2.7)	89 (1.0)	10 (0.1)	3758 (44.3)	8492
Codeine	1511 (22.5)	17 (0.3)	103 (1.5)	4 (0.1)	180 (2.7)	1931 (28.8)	314 (4.7)	69 (1.0)	2 (0.0)	2976 (44.4)	6706
Morphine	971 (26.6)	3 (0.1)	93 (2.6)	4 (0.1)	100 (2.7)	650 (17.8)	62 (1.7)	49 (1.3)	1 (0.0)	1861 (51.0)	3646
Aspirin	261 (14.5)	3 (0.2)	73 (4.1)	1 (0.1)	135 (7.5)	102 (5.7)	300 (16.7)	55 (3.1)	229 (12.7)	792 (44.0)	1800
Lisinopril	102 (6.4)	765 (48.1)	14 (0.9)	10 (0.6)	187 (11.8)	22 (1.4)	31 (1.9)	11 (0.7)	0 (0.0)	489 (30.7)	1591
Levofloxacin	464 (26.7)	1 (0.1)	43 (2.5)	54 (3.1)	122 (7.0)	138 (7.9)	77 (4.4)	13 (0.7)	3 (0.2)	903 (52.0)	1737
Erythromycin	420 (26.1)	1 (0.1)	24 (1.5)	5 (0.3)	55 (3.4)	306 (19.0)	281 (17.5)	18 (1.1)	3 (0.2)	611 (38.0)	1607
Meperidine	225 (15.0)	0 (0.0)	48 (3.2)	3 (0.2)	36 (2.4)	360 (24.0)	31 (2.1)	22 (1.5)	0 (0.0)	837 (55.8)	1499
Cephalexin	644 (44.1)	1 (0.1)	57 (3.9)	1 (0.1)	91 (6.2)	89 (6.1)	68 (4.7)	19 (1.3)	3 (0.2)	574 (39.3)	1460
Any statin	175 (6.0)	8 (0.3)	8 (0.3)	1504 (51.4)	55 (1.9)	84 (2.9)	133 (4.5)	10 (0.3)	4 (0.1)	1087 (37.2)	2924
Atorvastatin	50 (3.8)	5 (0.4)	2 (0.2)	657 (49.7)	25 (1.9)	35 (2.6)	52 (3.9)	5 (0.4)	2 (0.2)	526 (39.8)	1323
Simvastatin	52 (5.1)	1 (0.1)	2 (0.2)	496 (48.6)	11 (1.1)	18 (1.8)	37 (3.6)	1 (0.1)	1 (0.1)	422 (41.4)	1020
CYP2D6-metabolized opioids	2772 (27.0)	24 (0.2)	153 (1.5)	11 (0.1)	291 (2.8)	2883 (28.1)	456 (4.4)	115 (1.1)	5 (0.0)	4385 (42.7)	10264
African Ancestry											
Penicillin	887 (46.8)	0 (0.0)	84 (4.4)	0 (0.0)	181 (9.6)	65 (3.4)	48 (2.5)	6 (0.3)	2 (0.1)	731 (38.6)	1894
Sulfa	389 (40.4)	1 (0.1)	25 (2.6)	5 (0.5)	82 (8.5)	29 (3.0)	13 (1.3)	18 (1.9)	2 (0.2)	455 (47.2)	964
Codeine	195 (27.6)	2 (0.3)	7 (1.0)	0 (0.0)	34 (4.8)	144 (20.4)	38 (5.4)	13 (1.8)	0 (0.0)	313 (44.3)	706
Morphine	169 (37.6)	0 (0.0)	9 (2.0)	2 (0.4)	21 (4.7)	52 (11.6)	7 (1.6)	5 (1.1)	0 (0.0)	204 (45.3)	450
Aspirin	70 (17.5)	0 (0.0)	13 (3.2)	0 (0.0)	31 (7.7)	37 (9.2)	65 (16.2)	21 (5.2)	33 (8.2)	167 (41.6)	401
Lisinopril	22 (5.0)	136 (31.0)	9 (2.1)	5 (1.1)	157 (35.8)	5 (1.1)	6 (1.4)	4 (0.9)	0 (0.0)	107 (24.4)	439
Levofloxacin	37 (27.2)	0 (0.0)	3 (2.2)	3 (2.2)	13 (9.6)	7 (5.1)	6 (4.4)	4 (2.9)	0 (0.0)	66 (48.5)	136
Erythromycin	44 (31.9)	0 (0.0)	4 (2.9)	0 (0.0)	11 (8.0)	21 (15.2)	10 (7.2)	0 (0.0)	0 (0.0)	59 (42.8)	138
Meperidine	27 (24.1)	0 (0.0)	2 (1.8)	0 (0.0)	4 (3.6)	16 (14.3)	0 (0.0)	1 (0.9)	0 (0.0)	65 (58.0)	112
Cephalexin	56 (45.2)	0 (0.0)	4 (3.2)	0 (0.0)	8 (6.5)	7 (5.6)	7 (5.6)	1 (0.8)	0 (0.0)	46 (37.1)	124
Any statin	17 (6.6)	2 (0.8)	0 (0.0)	120 (46.7)	7 (2.7)	8 (3.1)	9 (3.5)	3 (1.2)	0 (0.0)	96 (37.4)	257
Atorvastatin	5 (5.8)	0 (0.0)	0 (0.0)	41 (47.7)	3 (3.5)	2 (2.3)	4 (4.7)	0 (0.0)	0 (0.0)	32 (37.2)	86
Simvastatin	10 (9.0)	1 (0.9)	0 (0.0)	46 (41.4)	0 (0.0)	5 (4.5)	4 (3.6)	2 (1.8)	0 (0.0)	44 (39.6)	111
CYP2D6-metabolized opioids	473 (35.2)	3 (0.2)	12 (0.9)	1 (0.1)	59 (4.4)	291 (21.7)	64 (4.8)	16 (1.2)	0 (0.0)	531 (39.5)	1343
