# Supplementary material for: High-throughput framework for genetic analyses of adverse drug reactions using electronic health records
Source: PLoS Genet. 2021 Jun 1;17(6):e1009593. doi: 10.1371/journal.pgen.1009593 (PMC8195357; doi:10.1371/journal.pgen.1009593)
Supplement: S2 Table — (PDF) [file pgen.1009593.s002.pdf]

S2 Table. Genome-wide significant variants associated with adverse drug reactions in self-reported European ancestry individuals.

ADVERSE DRUG REACTION	CHR	POS	SNP	FUNCTION	GENE	REF	ALT	EAF	R2	OR	SE	P
ASPIRIN	5	81062986	rs115346678	intergenic	SSBP2(dist=15914),ATG10(dist=204856)	G	A	0.010	0.985	2.031	0.125	1.40E-08
CEPHALEXIN	10	23704359	rs34545984	intergenic	LOC105376453(dist=70271),OTUD1(dist=23839)	G	T	0.015	0.504	2.040	0.125	1.23E-08
CODEINE	22	42405657	rs9620007	intronic	WBP2NL	C	G	0.301	0.984	0.837	0.024	1.24E-13
CODEINE	22	42389948	rs739296	intronic	SEPTIN3	G	A	0.301	0.988	0.838	0.024	1.57E-13
CODEINE	22	42388826	rs56234624	intronic	SEPTIN3	A	G	0.301	0.988	0.838	0.024	1.65E-13
CODEINE	22	42389431	rs2097561	intronic	SEPTIN3	T	C	0.301	0.988	0.838	0.024	1.65E-13
CODEINE	22	42389500	rs2097562	intronic	SEPTIN3	T	C	0.301	0.988	0.838	0.024	1.65E-13
CODEINE	22	42390209	rs7290907	intronic	SEPTIN3	G	A	0.301	0.989	0.838	0.024	1.66E-13
CODEINE	22	42390225	rs7290655	intronic	SEPTIN3	C	G	0.301	0.989	0.838	0.024	1.66E-13
CODEINE	22	42392811	rs1062753	UTR3	SEPTIN3(NM_019106:c.*2051G>A)	G	A	0.301	1.000	0.838	0.024	1.75E-13
CODEINE	22	42403980	rs59993856	intronic	WBP2NL	C	T	0.301	0.984	0.838	0.024	1.76E-13
CODEINE	22	42409977	rs9611710	intronic	WBP2NL	G	T	0.301	0.984	0.838	0.024	1.76E-13
CODEINE	22	42418110	rs9607869	intronic	WBP2NL	T	A	0.301	0.982	0.838	0.024	1.96E-13
CODEINE	22	42399547	rs9620006	intronic	WBP2NL	T	A	0.301	0.984	0.839	0.024	1.99E-13
CODEINE	22	42396371	rs8138080	intronic	WBP2NL	G	A	0.301	0.989	0.839	0.024	2.02E-13
CODEINE	22	42397026	rs9623489	intronic	WBP2NL	A	G	0.301	0.989	0.839	0.024	2.02E-13
CODEINE	22	42398266	rs9623490	intronic	WBP2NL	G	C	0.301	0.989	0.839	0.024	2.02E-13
CODEINE	22	42414181	rs9607868	intronic	WBP2NL	G	A	0.301	0.984	0.839	0.024	2.09E-13
CODEINE	22	42410419	rs12166549	intronic	WBP2NL	T	C	0.301	0.984	0.839	0.024	2.16E-13
CODEINE	22	42410695	rs9611711	intronic	WBP2NL	G	A	0.301	0.984	0.839	0.024	2.16E-13
CODEINE	22	42410993	rs9611712	intronic	WBP2NL	T	C	0.301	0.984	0.839	0.024	2.16E-13
CODEINE	22	42401514	rs9611700	intronic	WBP2NL	C	T	0.301	0.989	0.839	0.024	2.29E-13
CODEINE	22	42402399	rs2413663	intronic	WBP2NL	T	A	0.301	0.989	0.839	0.024	2.29E-13
CODEINE	22	42398195	rs11090066	intronic	WBP2NL	T	C	0.301	0.987	0.840	0.024	2.84E-13
CODEINE	22	42394612	rs4822079	upstream;downstream	WBP2NL(dist=180);SEPTIN3(dist=387)	G	C	0.301	0.984	0.840	0.024	3.13E-13
CODEINE	22	42632833	rs5758666	intronic	TCF20	G	A	0.286	0.985	0.840	0.024	5.07E-13
CODEINE	22	42633054	rs5751247	intronic	TCF20	T	C	0.285	0.984	0.840	0.024	5.82E-13
CODEINE	22	42620940	rs5751245	intronic	TCF20	T	C	0.288	0.987	0.843	0.024	1.21E-12
CODEINE	22	42406788	rs4822081	intronic	WBP2NL	C	T	0.304	0.976	0.845	0.024	1.98E-12
CODEINE	22	42614362	rs932376	intronic	TCF20	T	C	0.290	0.999	0.848	0.024	6.19E-12
CODEINE	22	42378507	rs62241000	intronic	SEPTIN3	C	T	0.321	0.989	0.853	0.023	1.08E-11
CODEINE	22	42378070	rs11914200	intronic	SEPTIN3	G	A	0.321	0.984	0.853	0.023	1.38E-11
CODEINE	22	42384171	rs11090065	intronic	SEPTIN3	T	C	0.322	0.986	0.855	0.023	2.03E-11
CODEINE	22	42386269	rs58874647	intronic	SEPTIN3	C	T	0.295	0.971	0.862	0.024	5.48E-10
CODEINE	22	42491479	rs5758579	ncRNA_intronic	NDUFA6-DT	T	C	0.210	0.961	0.850	0.027	1.90E-09
CODEINE	22	42492985	rs57117731	ncRNA_intronic	NDUFA6-DT	G	A	0.210	0.961	0.850	0.027	1.90E-09
CODEINE	22	42493875	rs5758580	ncRNA_intronic	NDUFA6-DT	T	C	0.210	0.961	0.850	0.027	1.90E-09
CODEINE	22	42486056	rs5751211	intronic	NDUFA6	G	A	0.210	0.959	0.852	0.027	2.88E-09
CODEINE	22	42488163	rs5758576	ncRNA_exonic	NDUFA6-DT	G	A	0.210	0.959	0.852	0.027	2.88E-09
CODEINE	22	42516366	rs5751221	ncRNA_intronic	NDUFA6-DT	C	T	0.211	0.958	0.852	0.027	3.10E-09
CODEINE	22	42517471	rs5758586	ncRNA_intronic	NDUFA6-DT	A	T	0.211	0.963	0.852	0.027	3.12E-09
CODEINE	22	42517922	rs5751222	ncRNA_intronic	NDUFA6-DT	T	A	0.211	0.963	0.852	0.027	3.12E-09
CODEINE	22	42453772	rs58099562	downstream	NAGA(dist=566)	T	C	0.199	0.947	0.849	0.028	3.23E-09
CODEINE	22	42511727	rs3985938	ncRNA_intronic	NDUFA6-DT	G	T	0.211	0.966	0.852	0.027	3.34E-09
CODEINE	22	42502070	rs9611734	ncRNA_intronic	NDUFA6-DT	A	G	0.211	0.983	0.853	0.027	3.91E-09
CODEINE	22	42472237	rs2413667	intronic	PHETA2	A	C	0.199	0.958	0.850	0.028	3.93E-09
CODEINE	22	42461918	rs2854827	intronic	NAGA	G	A	0.205	0.983	0.855	0.027	8.12E-09
CODEINE	22	42441091	rs28715885	intergenic	WBP2NL(dist=16614),NAGA(dist=13247)	T	G	0.195	0.946	0.857	0.028	2.86E-08
CODEINE	22	42471738	rs45441993	intronic	PHETA2	C	A	0.180	0.913	0.853	0.029	3.19E-08
CODEINE	22	42574555	rs5758629	intronic	TCF20	C	T	0.212	0.971	0.862	0.027	3.52E-08
CODEINE	22	42550287	rs1997659	intergenic	CYP2D7(dist=9803),TCF20(dist=5732)	A	G	0.217	0.840	0.864	0.027	4.07E-08
CODEINE	22	42563308	rs4453786	intronic	TCF20	C	T	0.212	0.998	0.863	0.027	4.13E-08
CODEINE	22	42563529	rs62240863	intronic	TCF20	T	G	0.209	0.926	0.862	0.027	4.41E-08
CODEINE	22	42573139	rs1989375	intronic	TCF20	C	G	0.215	0.972	0.864	0.027	4.55E-08
CODEINE	22	42436322	rs9607871	intergenic	WBP2NL(dist=11845),NAGA(dist=18016)	G	A	0.198	0.955	0.860	0.028	4.72E-08
CODEINE	22	42564304	rs5751232	intronic	TCF20	C	T	0.211	0.988	0.863	0.027	4.80E-08
CODEINE	22	42563933	rs5758622	intronic	TCF20	C	T	0.211	0.988	0.863	0.027	4.82E-08
CODEINE	22	42359302	rs1984419	intergenic	LINC00634(dist=4356),SEPTIN3(dist=6140)	T	C	0.347	0.984	0.883	0.023	4.92E-08
CYP2D6-METABOLIZED OPIOIDS	6	154390607	rs62436463	intronic	OPRM1	C	T	0.102	0.941	0.844	0.027	5.43E-10
CYP2D6-METABOLIZED OPIOIDS	6	154360797	rs1799971	exonic	OPRM1	A	G	0.131	0.999	0.864	0.024	2.42E-09
CYP2D6-METABOLIZED OPIOIDS	6	154335673	rs1294092	intronic	OPRM1	A	G	0.165	0.988	0.879	0.022	5.73E-09
CYP2D6-METABOLIZED OPIOIDS	6	154337179	rs1294088	intronic	OPRM1	T	C	0.165	0.986	0.879	0.022	5.93E-09
CYP2D6-METABOLIZED OPIOIDS	6	154337784	rs1294086	intronic	OPRM1	A	G	0.165	0.985	0.880	0.022	7.08E-09
CYP2D6-METABOLIZED OPIOIDS	6	154336426	rs1294091	intronic	OPRM1	A	G	0.165	0.984	0.880	0.022	7.47E-09
CYP2D6-METABOLIZED OPIOIDS	22	42389948	rs739296	intronic	SEPTIN3	G	A	0.301	0.988	0.863	0.018	1.08E-16
CYP2D6-METABOLIZED OPIOIDS	22	42388826	rs56234624	intronic	SEPTIN3	A	G	0.301	0.988	0.863	0.018	1.17E-16
CYP2D6-METABOLIZED OPIOIDS	22	42389431	rs2097561	intronic	SEPTIN3	T	C	0.301	0.988	0.863	0.018	1.17E-16
CYP2D6-METABOLIZED OPIOIDS	22	42389500	rs2097562	intronic	SEPTIN3	T	C	0.301	0.988	0.863	0.018	1.17E-16
CYP2D6-METABOLIZED OPIOIDS	22	42392811	rs1062753	UTR3	SEPTIN3(NM_019106:c.*2051G>A)	G	A	0.301	1.000	0.863	0.018	1.17E-16
CYP2D6-METABOLIZED OPIOIDS	22	42390209	rs7290907	intronic	SEPTIN3	G	A	0.301	0.989	0.863	0.018	1.19E-16
CYP2D6-METABOLIZED OPIOIDS	22	42390225	rs7290655	intronic	SEPTIN3	C	G	0.301	0.989	0.863	0.018	1.19E-16
CYP2D6-METABOLIZED OPIOIDS	22	42405657	rs9620007	intronic	WBP2NL	C	G	0.301	0.984	0.863	0.018	1.20E-16
CYP2D6-METABOLIZED OPIOIDS	22	42396371	rs8138080	intronic	WBP2NL	G	A	0.301	0.989	0.864	0.018	1.56E-16
CYP2D6-METABOLIZED OPIOIDS	22	42397026	rs9623489	intronic	WBP2NL	A	G	0.301	0.989	0.864	0.018	1.56E-16
CYP2D6-METABOLIZED OPIOIDS	22	42398266	rs9623490	intronic	WBP2NL	G	C	0.301	0.989	0.864	0.018	1.56E-16
CYP2D6-METABOLIZED OPIOIDS	22	42399547	rs9620006	intronic	WBP2NL	T	A	0.301	0.984	0.864	0.018	1.62E-16
CYP2D6-METABOLIZED OPIOIDS	22	42401514	rs9611700	intronic	WBP2NL	C	T	0.301	0.989	0.864	0.018	1.72E-16
CYP2D6-METABOLIZED OPIOIDS	22	42402399	rs2413663	intronic	WBP2NL	T	A	0.301	0.989	0.864	0.018	1.72E-16
CYP2D6-METABOLIZED OPIOIDS	22	42398195	rs11090066	intronic	WBP2NL	T	C	0.301	0.987	0.864	0.018	1.77E-16
CYP2D6-METABOLIZED OPIOIDS	22	42418110	rs9607869	intronic	WBP2NL	T	A	0.301	0.982	0.864	0.018	1.85E-16
CYP2D6-METABOLIZED OPIOIDS	22	42403980	rs59993856	intronic	WBP2NL	C	T	0.301	0.984	0.864	0.018	1.85E-16
CYP2D6-METABOLIZED OPIOIDS	22	42409977	rs9611710	intronic	WBP2NL	G	T	0.301	0.984	0.864	0.018	1.85E-16
CYP2D6-METABOLIZED OPIOIDS	22	42410419	rs12166549	intronic	WBP2NL	T	C	0.301	0.984	0.864	0.018	2.27E-16
CYP2D6-METABOLIZED OPIOIDS	22	42410695	rs9611711	intronic	WBP2NL	G	A	0.301	0.984	0.864	0.018	2.27E-16
CYP2D6-METABOLIZED OPIOIDS	22	42410993	rs9611712	intronic	WBP2NL	T	C	0.301	0.984	0.864	0.018	2.27E-16
CYP2D6-METABOLIZED OPIOIDS	22	42394612	rs4822079	upstream;downstream	WBP2NL(dist=180);SEPTIN3(dist=387)	G	C	0.301	0.984	0.864	0.018	2.30E-16
CYP2D6-METABOLIZED OPIOIDS	22	42414181	rs9607868	intronic	WBP2NL	G	A	0.301	0.984	0.865	0.018	2.40E-16
CYP2D6-METABOLIZED OPIOIDS	22	42406788	rs4822081	intronic	WBP2NL	C	T	0.304	0.976	0.867	0.018	6.75E-16
CYP2D6-METABOLIZED OPIOIDS	22	42453772	rs58099562	downstream	NAGA(dist=566)	T	C	0.199	0.947	0.856	0.020	3.11E-14
CYP2D6-METABOLIZED OPIOIDS	22	42472237	rs2413667	intronic	PHETA2	A	C	0.199	0.958	0.856	0.020	3.49E-14
CYP2D6-METABOLIZED OPIOIDS	22	42633054	rs5751247	intronic	TCF20	T	C	0.285	0.984	0.874	0.018	6.31E-14
CYP2D6-METABOLIZED OPIOIDS	22	42493875	rs5758580	ncRNA_intronic	NDUFA6-DT	T	C	0.210	0.961	0.861	0.020	6.79E-14
CYP2D6-METABOLIZED OPIOIDS	22	42492985	rs57117731	ncRNA_intronic	NDUFA6-DT	G	A	0.210	0.961	0.861	0.020	6.97E-14
CYP2D6-METABOLIZED OPIOIDS	22	42632833	rs5758666	intronic	TCF20	G	A	0.286	0.985	0.874	0.018	7.14E-14
CYP2D6-METABOLIZED OPIOIDS	22	42491479	rs5758579	ncRNA_intronic	NDUFA6-DT	T	C	0.210	0.961	0.861	0.020	7.47E-14
CYP2D6-METABOLIZED OPIOIDS	22	42441091	rs28715885	intergenic	WBP2NL(dist=16614),NAGA(dist=13247)	T	G	0.195	0.946	0.857	0.021	7.93E-14
CYP2D6-METABOLIZED OPIOIDS	22	42502070	rs9611734	ncRNA_intronic	NDUFA6-DT	A	G	0.211	0.983	0.861	0.020	8.27E-14
CYP2D6-METABOLIZED OPIOIDS	22	42516366	rs5751221	ncRNA_intronic	NDUFA6-DT	C	T	0.211	0.958	0.862	0.020	1.01E-13
CYP2D6-METABOLIZED OPIOIDS	22	42517471	rs5758586	ncRNA_intronic	NDUFA6-DT	A	T	0.211	0.963	0.862	0.020	1.01E-13
CYP2D6-METABOLIZED OPIOIDS	22	42517922	rs5751222	ncRNA_intronic	NDUFA6-DT	T	A	0.211	0.963	0.862	0.020	1.01E-13
CYP2D6-METABOLIZED OPIOIDS	22	42511727	rs3985938	ncRNA_intronic	NDUFA6-DT	G	T	0.211	0.966	0.862	0.020	1.07E-13

CYP2D6-METABOLIZED OPIOIDS	22	42488163	rs5758576	ncRNA_exonic	NDUFA6-DT	G	A	0.210	0.959	0.862	0.020	1.21E-13
CYP2D6-METABOLIZED OPIOIDS	22	42486056	rs5751211	intronic	NDUFA6	G	A	0.210	0.959	0.862	0.020	1.25E-13
CYP2D6-METABOLIZED OPIOIDS	22	42461918	rs2854827	intronic	NAGA	G	A	0.205	0.983	0.861	0.020	1.34E-13
CYP2D6-METABOLIZED OPIOIDS	22	42563529	rs62240863	intronic	TCF20	T	G	0.209	0.926	0.862	0.020	1.69E-13
CYP2D6-METABOLIZED OPIOIDS	22	42620940	rs5751245	intronic	TCF20	T	C	0.288	0.987	0.877	0.018	1.69E-13
CYP2D6-METABOLIZED OPIOIDS	22	42436322	rs9607871	intergenic	WBP2NL(dist=11845),NAGA(dist=18016)	G	A	0.198	0.955	0.860	0.021	2.13E-13
CYP2D6-METABOLIZED OPIOIDS	22	42550287	rs1997659	intergenic	CYP2D7(dist=9803),TCF20(dist=5732)	A	G	0.217	0.840	0.865	0.020	2.27E-13
CYP2D6-METABOLIZED OPIOIDS	22	42554839	rs5758618	intergenic	CYP2D7(dist=14355),TCF20(dist=1180)	C	T	0.216	0.979	0.865	0.020	3.16E-13
CYP2D6-METABOLIZED OPIOIDS	22	42574555	rs5758629	intronic	TCF20	C	T	0.212	0.971	0.865	0.020	3.49E-13
CYP2D6-METABOLIZED OPIOIDS	22	42554409	rs2899354	intergenic	CYP2D7(dist=13925),TCF20(dist=1610)	G	T	0.212	0.981	0.865	0.020	3.58E-13
CYP2D6-METABOLIZED OPIOIDS	22	42549164	rs5758613	intergenic	CYP2D7(dist=8680),TCF20(dist=6855)	A	G	0.217	0.838	0.866	0.020	3.61E-13
CYP2D6-METABOLIZED OPIOIDS	22	42551344	rs2236779	intergenic	CYP2D7(dist=10860),TCF20(dist=4675)	G	A	0.214	0.977	0.866	0.020	4.14E-13
CYP2D6-METABOLIZED OPIOIDS	22	42548094	rs5758611	intergenic	CYP2D7(dist=7610),TCF20(dist=7925)	A	G	0.216	0.819	0.866	0.020	4.51E-13
CYP2D6-METABOLIZED OPIOIDS	22	42563308	rs4453786	intronic	TCF20	C	T	0.212	0.998	0.866	0.020	4.89E-13
CYP2D6-METABOLIZED OPIOIDS	22	42573139	rs1989375	intronic	TCF20	C	G	0.215	0.972	0.866	0.020	5.25E-13
CYP2D6-METABOLIZED OPIOIDS	22	42572547	rs5758627	intronic	TCF20	C	T	0.211	0.977	0.866	0.020	5.30E-13
CYP2D6-METABOLIZED OPIOIDS	22	42555861	rs5751231	downstream	TCF20(dist=158)	T	A	0.217	0.980	0.867	0.020	5.55E-13
CYP2D6-METABOLIZED OPIOIDS	22	42578596	rs9607885	intronic	TCF20	T	C	0.214	0.975	0.866	0.020	5.64E-13
CYP2D6-METABOLIZED OPIOIDS	22	42572363	rs5758626	intronic	TCF20	G	A	0.211	0.976	0.866	0.020	6.00E-13
CYP2D6-METABOLIZED OPIOIDS	22	42564304	rs5751232	intronic	TCF20	C	T	0.211	0.988	0.866	0.020	6.04E-13
CYP2D6-METABOLIZED OPIOIDS	22	42567451	rs9607882	intronic	TCF20	A	G	0.211	0.986	0.866	0.020	6.04E-13
CYP2D6-METABOLIZED OPIOIDS	22	42471738	rs45441993	intronic	PHETA2	C	A	0.180	0.913	0.858	0.021	6.31E-13
CYP2D6-METABOLIZED OPIOIDS	22	42563933	rs5758622	intronic	TCF20	C	T	0.211	0.988	0.866	0.020	6.39E-13
CYP2D6-METABOLIZED OPIOIDS	22	42614362	rs932376	intronic	TCF20	T	C	0.290	0.999	0.880	0.018	6.40E-13
CYP2D6-METABOLIZED OPIOIDS	22	42628335	rs56906457	intronic	TCF20	A	G	0.188	0.999	0.860	0.021	6.54E-13
CYP2D6-METABOLIZED OPIOIDS	22	42569298	rs738257	intronic	TCF20	C	A	0.211	0.984	0.866	0.020	6.67E-13
CYP2D6-METABOLIZED OPIOIDS	22	42580933	rs5758637	intronic	TCF20	A	C	0.216	0.969	0.867	0.020	6.98E-13
CYP2D6-METABOLIZED OPIOIDS	22	42579923	rs5758635	intronic	TCF20	T	C	0.215	0.972	0.867	0.020	7.50E-13
CYP2D6-METABOLIZED OPIOIDS	22	42553353	rs5751230	intergenic	CYP2D7(dist=12869),TCF20(dist=2666)	G	A	0.215	0.978	0.868	0.020	8.48E-13
CYP2D6-METABOLIZED OPIOIDS	22	42582394	rs5758638	intronic	TCF20	T	C	0.216	0.971	0.868	0.020	1.03E-12
CYP2D6-METABOLIZED OPIOIDS	22	42548178	rs5758612	intergenic	CYP2D7(dist=7694),TCF20(dist=7841)	A	G	0.219	0.828	0.869	0.020	1.12E-12
CYP2D6-METABOLIZED OPIOIDS	22	42547755	rs8137366	intergenic	CYP2D7(dist=7271),TCF20(dist=8264)	C	G	0.219	0.826	0.869	0.020	1.17E-12
CYP2D6-METABOLIZED OPIOIDS	22	42569870	rs713811	intronic	TCF20	C	T	0.220	0.976	0.870	0.020	1.41E-12
CYP2D6-METABOLIZED OPIOIDS	22	42569999	rs2011944	intronic	TCF20	C	T	0.218	0.980	0.869	0.020	1.45E-12
CYP2D6-METABOLIZED OPIOIDS	22	42644471	rs55906806	intronic	TCF20	C	T	0.186	0.992	0.862	0.021	1.52E-12
CYP2D6-METABOLIZED OPIOIDS	22	42569024	rs714002	intronic	TCF20	T	C	0.219	0.978	0.870	0.020	1.53E-12
CYP2D6-METABOLIZED OPIOIDS	22	42547409	rs5758610	intergenic	CYP2D7(dist=6925),TCF20(dist=8610)	C	T	0.214	0.788	0.869	0.020	1.59E-12
CYP2D6-METABOLIZED OPIOIDS	22	42547229	rs5758608	intergenic	CYP2D7(dist=6745),TCF20(dist=8790)	C	T	0.214	0.788	0.869	0.020	1.59E-12
CYP2D6-METABOLIZED OPIOIDS	22	42547317	rs5758609	intergenic	CYP2D7(dist=6833),TCF20(dist=8702)	A	G	0.214	0.788	0.869	0.020	1.59E-12
CYP2D6-METABOLIZED OPIOIDS	22	42561365	rs9611746	intronic	TCF20	A	G	0.213	0.983	0.869	0.020	1.69E-12
CYP2D6-METABOLIZED OPIOIDS	22	42654327	rs17478227	intronic	TCF20	C	G	0.185	0.993	0.863	0.021	1.92E-12
CYP2D6-METABOLIZED OPIOIDS	22	42563889	rs62240864	intronic	TCF20	C	T	0.185	0.977	0.863	0.021	2.16E-12
CYP2D6-METABOLIZED OPIOIDS	22	42633204	rs5758667	intronic	TCF20	G	A	0.200	0.987	0.868	0.020	3.27E-12
CYP2D6-METABOLIZED OPIOIDS	22	42655377	rs5758686	intronic	TCF20	G	T	0.200	0.988	0.868	0.020	4.04E-12
CYP2D6-METABOLIZED OPIOIDS	22	42648353	rs34107327	intronic	TCF20	T	G	0.200	0.986	0.868	0.020	4.10E-12
CYP2D6-METABOLIZED OPIOIDS	22	42642576	rs58654759	intronic	TCF20	T	C	0.200	0.985	0.868	0.020	4.19E-12
CYP2D6-METABOLIZED OPIOIDS	22	42662501	rs5758688	intronic	TCF20	T	C	0.199	0.985	0.868	0.020	4.52E-12
CYP2D6-METABOLIZED OPIOIDS	22	42645202	rs5758682	intronic	TCF20	C	T	0.200	0.988	0.868	0.020	4.59E-12
CYP2D6-METABOLIZED OPIOIDS	22	42684818	rs5758698	intergenic	TCF20(dist=4885),LINC01315(dist=75588)	C	T	0.198	0.920	0.868	0.020	4.73E-12
CYP2D6-METABOLIZED OPIOIDS	22	42667473	rs1107553	ncRNA_intronic	OGFRP1	A	G	0.200	0.977	0.869	0.020	5.13E-12
CYP2D6-METABOLIZED OPIOIDS	22	42378507	rs62241000	intronic	SEPTIN3	C	T	0.321	0.989	0.887	0.017	5.22E-12
CYP2D6-METABOLIZED OPIOIDS	22	42628088	rs5758662	intronic	TCF20	T	C	0.200	0.986	0.869	0.020	5.26E-12
CYP2D6-METABOLIZED OPIOIDS	22	42667594	rs1107554	ncRNA_intronic	OGFRP1	G	C	0.199	0.981	0.869	0.020	5.42E-12
CYP2D6-METABOLIZED OPIOIDS	22	42664972	rs5758689	intronic	TCF20	T	G	0.200	0.979	0.869	0.020	5.50E-12
CYP2D6-METABOLIZED OPIOIDS	22	42645091	rs5758681	intronic	TCF20	A	G	0.201	0.987	0.869	0.020	5.96E-12
CYP2D6-METABOLIZED OPIOIDS	22	42663871	rs5751258	intronic	TCF20	C	G	0.200	0.983	0.869	0.020	5.97E-12
CYP2D6-METABOLIZED OPIOIDS	22	42618669	rs5758657	intronic	TCF20	A	G	0.200	0.990	0.869	0.020	6.46E-12
CYP2D6-METABOLIZED OPIOIDS	22	42619067	rs1033459	intronic	TCF20	A	G	0.200	0.990	0.869	0.020	6.46E-12
CYP2D6-METABOLIZED OPIOIDS	22	42651536	rs56400210	intronic	TCF20	A	C	0.187	0.995	0.866	0.021	6.49E-12
CYP2D6-METABOLIZED OPIOIDS	22	42595258	rs5751240	intronic	TCF20	G	C	0.216	0.962	0.873	0.020	6.66E-12
CYP2D6-METABOLIZED OPIOIDS	22	42660414	rs739146	intronic	TCF20	A	G	0.201	0.986	0.870	0.020	6.80E-12
CYP2D6-METABOLIZED OPIOIDS	22	42612408	rs5758652	intronic	TCF20	T	C	0.200	0.990	0.870	0.020	6.81E-12
CYP2D6-METABOLIZED OPIOIDS	22	42666069	rs55644935	ncRNA_intronic	OGFRP1	A	G	0.201	0.980	0.870	0.020	6.91E-12
CYP2D6-METABOLIZED OPIOIDS	22	42649509	rs5758684	intronic	TCF20	C	T	0.200	0.986	0.870	0.020	7.18E-12
CYP2D6-METABOLIZED OPIOIDS	22	42609148	rs5758651	exonic	TCF20	T	C	0.200	0.987	0.870	0.020	7.28E-12
CYP2D6-METABOLIZED OPIOIDS	22	42378070	rs11914200	intronic	SEPTIN3	G	A	0.321	0.984	0.888	0.017	7.37E-12
CYP2D6-METABOLIZED OPIOIDS	22	42656588	rs5758687	intronic	TCF20	A	T	0.201	0.984	0.870	0.020	7.54E-12
CYP2D6-METABOLIZED OPIOIDS	22	42619308	rs1033460	intronic	TCF20	A	G	0.200	0.991	0.870	0.020	7.59E-12
CYP2D6-METABOLIZED OPIOIDS	22	42623258	rs55867855	intronic	TCF20	G	A	0.200	0.991	0.870	0.020	7.59E-12
CYP2D6-METABOLIZED OPIOIDS	22	42668504	rs5758691	ncRNA_intronic	OGFRP1	G	C	0.199	0.965	0.870	0.020	7.71E-12
CYP2D6-METABOLIZED OPIOIDS	22	42688532	rs17002947	intergenic	TCF20(dist=8599),LINC01315(dist=71874)	A	G	0.187	0.914	0.866	0.021	7.79E-12
CYP2D6-METABOLIZED OPIOIDS	22	42384171	rs11090065	intronic	SEPTIN3	T	C	0.322	0.986	0.888	0.017	7.93E-12
CYP2D6-METABOLIZED OPIOIDS	22	42669029	rs5758692	ncRNA_intronic	OGFRP1	T	A	0.199	0.962	0.870	0.020	8.00E-12
CYP2D6-METABOLIZED OPIOIDS	22	42670293	rs1001586	ncRNA_exonic	OGFRP1	G	T	0.199	0.966	0.870	0.020	8.00E-12
CYP2D6-METABOLIZED OPIOIDS	22	42668295	rs5758690	ncRNA_intronic	OGFRP1	C	G	0.200	0.982	0.870	0.020	8.21E-12
CYP2D6-METABOLIZED OPIOIDS	22	42671066	rs739147	intronic	TCF20	T	G	0.199	0.965	0.870	0.020	8.34E-12
CYP2D6-METABOLIZED OPIOIDS	22	42664883	rs56111723	intronic	TCF20	T	A	0.201	0.984	0.870	0.020	8.99E-12
CYP2D6-METABOLIZED OPIOIDS	22	42670111	rs1001587	ncRNA_exonic	OGFRP1	C	T	0.200	0.965	0.872	0.020	1.66E-11
CYP2D6-METABOLIZED OPIOIDS	22	42617177	rs5751243	intronic	TCF20	T	C	0.202	0.989	0.872	0.020	1.74E-11
CYP2D6-METABOLIZED OPIOIDS	22	42617411	rs5751244	intronic	TCF20	T	C	0.202	0.989	0.872	0.020	1.74E-11
CYP2D6-METABOLIZED OPIOIDS	22	42521985	rs4078247	downstream	CYP2D6,LOC101929829,NDUFA6-DT(dist=516)	T	C	0.196	0.711	0.871	0.021	2.21E-11
CYP2D6-METABOLIZED OPIOIDS	22	42522392	rs28371738	downstream	CYP2D6,LOC101929829(dist=109)	G	A	0.196	0.708	0.872	0.021	2.31E-11
CYP2D6-METABOLIZED OPIOIDS	22	42688034	rs5758699	intergenic	TCF20(dist=8101),LINC01315(dist=72372)	C	G	0.203	0.916	0.876	0.020	6.67E-11
CYP2D6-METABOLIZED OPIOIDS	22	42544551	rs9607880	intergenic	CYP2D7(dist=4067),TCF20(dist=11468)	C	G	0.147	0.652	0.875	0.023	7.22E-09
CYP2D6-METABOLIZED OPIOIDS	22	42385429	rs62241023	intronic	SEPTIN3	G	A	0.226	0.972	0.894	0.019	7.91E-09
CYP2D6-METABOLIZED OPIOIDS	22	42386269	rs58874647	intronic	SEPTIN3	C	T	0.295	0.971	0.903	0.018	8.14E-09
CYP2D6-METABOLIZED OPIOIDS	22	42365073	rs10154700	upstream	SEPTIN3(dist=369)	G	T	0.221	0.976	0.896	0.020	1.82E-08
CYP2D6-METABOLIZED OPIOIDS	22	42359302	rs1984419	intergenic	LINC00634(dist=4356),SEPTIN3(dist=6140)	T	C	0.347	0.984	0.909	0.017	2.06E-08
CYP2D6-METABOLIZED OPIOIDS	22	42344911	rs62240992	intergenic	CENPM(dist=1743),LINC00634(dist=3280)	A	G	0.221	0.992	0.897	0.020	2.36E-08
CYP2D6-METABOLIZED OPIOIDS	22	42356430	rs7288749	intergenic	LINC00634(dist=1484),SEPTIN3(dist=9012)	C	T	0.223	0.991	0.897	0.019	2.63E-08
CYP2D6-METABOLIZED OPIOIDS	22	42351289	rs12158579	ncRNA_intronic	LINC00634	C	T	0.228	0.992	0.898	0.019	2.85E-08
CYP2D6-METABOLIZED OPIOIDS	22	42351477	rs9680671	ncRNA_intronic	LINC00634	T	C	0.228	0.992	0.898	0.019	2.99E-08
CYP2D6-METABOLIZED OPIOIDS	22	42344297	rs8140869	intergenic	CENPM(dist=1129),LINC00634(dist=3894)	G	A	0.224	0.999	0.898	0.019	3.08E-08
CYP2D6-METABOLIZED OPIOIDS	22	42354389	rs62240996	ncRNA_exonic	LINC00634	A	C	0.356	0.999	0.911	0.017	3.39E-08
CYP2D6-METABOLIZED OPIOIDS	22	42355549	rs73165164	downstream	LINC00634(dist=603)	T	C	0.224	0.991	0.898	0.019	3.40E-08
CYP2D6-METABOLIZED OPIOIDS	22	42354567	rs62240997	ncRNA_exonic	LINC00634	T	G	0.224	0.992	0.898	0.019	3.41E-08
CYP2D6-METABOLIZED OPIOIDS	22	42349134	rs1894714	ncRNA_intronic	LINC00634	T	G	0.776	0.972	1.113	0.019	3.58E-08
CYP2D6-METABOLIZED OPIOIDS	22	42349710	rs5751186	ncRNA_intronic	LINC00634	G	A	0.776	0.972	1.113	0.019	3.58E-08
CYP2D6-METABOLIZED OPIOIDS	22	42355563	rs62240999	downstream	LINC00634(dist=617)	C	G	0.224	0.991	0.898	0.019	3.68E-08
CYP2D6-METABOLIZED OPIOIDS	22	42346781	rs55651128	intergenic	CENPM(dist=3613),LINC00634(dist=1410)	T	C	0.224	0.992	0.898	0.019	3.83E-08
CYP2D6-METABOLIZED OPIOIDS	22	42347371	rs11913631	upstream	LINC00634(dist=820)	G	T	0.224	0.992	0.898	0.019	3.83E-08
CYP2D6-METABOLIZED OPIOIDS	22	42347391	rs11913578	upstream	LINC00634(dist=800)	A	C	0.224	0.992	0.899	0.019	3.98E-08
CYP2D6-METABOLIZED OPIOIDS	22	42350127	rs55718937	ncRNA_intronic	LINC00634	G	A	0.224	0.992	0.899	0.019	3.98E-08

CYP2D6-METABOLIZED OPIOIDS	22	42351309	rs58302269	ncRNA_intronic	LINC00634	G	A	0.224	0.989	0.899	0.019	3.99E-08
CYP2D6-METABOLIZED OPIOIDS	22	42346475	rs12167978	intergenic	CENPM(dist=3307),LINC00634(dist=1716)	G	A	0.224	0.992	0.899	0.019	4.05E-08
CYP2D6-METABOLIZED OPIOIDS	22	42345181	rs5751182	intergenic	CENPM(dist=2013),LINC00634(dist=3010)	G	A	0.643	0.980	1.097	0.017	4.15E-08
CYP2D6-METABOLIZED OPIOIDS	22	42356886	rs6002560	intergenic	LINC00634(dist=1940),SEPTIN3(dist=8556)	T	C	0.355	1.000	0.912	0.017	4.41E-08
CYP2D6-METABOLIZED OPIOIDS	22	42360574	rs9623482	intergenic	LINC00634(dist=5628),SEPTIN3(dist=4868)	T	C	0.224	0.985	0.899	0.019	4.98E-08
MEPERIDINE	12	28161055	rs11049274	intergenic	PTHLH(dist=35389),LOC729291(dist=177503)	G	A	0.081	0.999	1.419	0.062	2.09E-08
MEPERIDINE	12	28162341	rs11049275	intergenic	PTHLH(dist=36675),LOC729291(dist=176217)	T	A	0.081	0.999	1.419	0.062	2.09E-08
MEPERIDINE	12	28171821	rs10843055	intergenic	PTHLH(dist=46155),LOC729291(dist=166737)	A	C	0.081	0.971	1.418	0.062	2.19E-08
MEPERIDINE	12	28177171	rs11049291	intergenic	PTHLH(dist=51505),LOC729291(dist=161387)	A	G	0.081	0.952	1.418	0.062	2.19E-08
MEPERIDINE	4	54287697	rs113100019		FIP1L1	T	G	0.012	0.822	2.101	0.133	2.26E-08
MEPERIDINE	5	79480369	rs185462714	intronic	SERINC5	A	C	0.011	0.821	2.087	0.133	3.37E-08
MEPERIDINE	5	79480371	rs191012979	intronic	SERINC5	A	G	0.011	0.821	2.087	0.133	3.37E-08
MEPERIDINE	12	28145898	rs11049260	intergenic	PTHLH(dist=20232),LOC729291(dist=192660)	A	T	0.081	1.000	1.409	0.063	4.16E-08
PENICILLIN	6	31327622	rs115200108	intergenic	HLA-B(dist=2666),MICA-AS1(dist=34444)	C	A	0.025	0.999	1.302	0.045	4.23E-09
PENICILLIN	6	31340628	rs72878037	intergenic	HLA-B(dist=15672),MICA-AS1(dist=21438)	C	T	0.025	0.992	1.295	0.044	5.75E-09
PENICILLIN	6	31340795	rs72878039	intergenic	HLA-B(dist=15839),MICA-AS1(dist=21271)	A	G	0.025	0.992	1.295	0.044	5.75E-09
PENICILLIN	6	31344484	rs114654060	intergenic	HLA-B(dist=19528),MICA-AS1(dist=17582)	C	T	0.025	0.999	1.297	0.045	6.65E-09
PENICILLIN	6	31344485	rs116355076	intergenic	HLA-B(dist=19529),MICA-AS1(dist=17581)	G	T	0.025	1.000	1.297	0.045	6.65E-09
PENICILLIN	6	31348200	rs4143334	intergenic	HLA-B(dist=23244),MICA-AS1(dist=13866)	A	G	0.025	1.000	1.296	0.045	6.73E-09
PENICILLIN	6	31351321	rs72865324	intergenic	HLA-B(dist=26365),MICA-AS1(dist=10745)	C	T	0.025	1.000	1.297	0.045	6.76E-09
PENICILLIN	6	31336558	rs60177449	intergenic	HLA-B(dist=11602),MICA-AS1(dist=25508)	G	A	0.025	0.998	1.297	0.045	6.91E-09
PENICILLIN	6	31334799	rs7766461	intergenic	HLA-B(dist=9843),MICA-AS1(dist=27267)	C	T	0.025	0.997	1.296	0.045	7.54E-09
PENICILLIN	6	31353285	rs72882965	intergenic	HLA-B(dist=28329),MICA-AS1(dist=8781)	T	C	0.025	0.999	1.296	0.045	7.55E-09
PENICILLIN	6	31355813	rs2428484	intergenic	HLA-B(dist=30857),MICA-AS1(dist=6253)	C	G	0.025	0.999	1.295	0.045	7.82E-09
PENICILLIN	6	31325201	rs74194187	upstream	HLA-B(dist=245)	C	A	0.025	0.999	1.293	0.045	8.70E-09
PENICILLIN	6	31326140	rs114492969	intergenic	HLA-B(dist=1184),MICA-AS1(dist=35926)	C	T	0.025	0.999	1.293	0.045	8.70E-09
PENICILLIN	6	31326312	rs145887584	intergenic	HLA-B(dist=1356),MICA-AS1(dist=35754)	C	A	0.025	0.999	1.293	0.045	8.70E-09
PENICILLIN	6	31412306	rs55857629	ncRNA_intronic	LINC01149	A	G	0.025	0.999	1.294	0.045	9.35E-09
PENICILLIN	6	31324941	rs114038515	UTR5	HLA-B(NM_005514:c.-6G>A)	C	T	0.025	0.977	1.296	0.045	1.00E-08
PENICILLIN	6	31379059	rs3819268	exonic	MICA	A	T	0.025	0.998	1.293	0.045	1.02E-08
PENICILLIN	6	31386599	rs114905838	intergenic	MICA(dist=3507),LINC01149(dist=22845)	T	C	0.025	0.998	1.293	0.045	1.02E-08
PENICILLIN	6	31388555	rs72847395	intergenic	MICA(dist=5463),LINC01149(dist=20889)	T	G	0.025	0.999	1.293	0.045	1.02E-08
PENICILLIN	6	31376358	rs56370321	intronic	MICA	C	A	0.025	0.996	1.293	0.045	1.07E-08
PENICILLIN	6	31322767	rs3819284	intronic	HLA-B	G	A	0.025	0.999	1.291	0.045	1.10E-08
PENICILLIN	6	31323414	rs41563818	intronic	HLA-B	G	A	0.025	0.999	1.291	0.045	1.10E-08
PENICILLIN	6	31323707	rs41545339	intronic	HLA-B	G	A	0.025	0.999	1.291	0.045	1.10E-08
PENICILLIN	6	31377558	rs72502581	intronic	MICA	G	A	0.025	0.996	1.293	0.045	1.10E-08
PENICILLIN	6	31414673	rs72851111	ncRNA_exonic	LINC01149	G	A	0.025	0.999	1.293	0.045	1.11E-08
PENICILLIN	6	31426427	rs72851199	intergenic	LINC01149(dist=11677),HCP5(dist=4530)	G	A	0.025	0.999	1.292	0.045	1.14E-08
PENICILLIN	6	31426642	rs72841203	intergenic	LINC01149(dist=11892),HCP5(dist=4315)	T	A	0.025	0.999	1.292	0.045	1.14E-08
PENICILLIN	6	31426643	rs115427390	intergenic	LINC01149(dist=11893),HCP5(dist=4314)	G	C	0.025	0.999	1.292	0.045	1.14E-08
PENICILLIN	6	31394967	rs149304476	intergenic	MICA(dist=11875),LINC01149(dist=14477)	G	C	0.025	0.998	1.292	0.045	1.16E-08
PENICILLIN	6	31395052	rs72848713	intergenic	MICA(dist=11960),LINC01149(dist=14392)	G	C	0.025	0.998	1.292	0.045	1.16E-08
PENICILLIN	6	31416912	rs56241874	intergenic	LINC01149(dist=2162),HCP5(dist=14045)	A	C	0.025	0.999	1.292	0.045	1.18E-08
PENICILLIN	6	31416956	rs56180073	intergenic	LINC01149(dist=2206),HCP5(dist=14001)	C	T	0.025	0.999	1.292	0.045	1.18E-08
PENICILLIN	6	31417249	rs55691135	intergenic	LINC01149(dist=2499),HCP5(dist=13708)	G	A	0.025	0.999	1.292	0.045	1.18E-08
PENICILLIN	6	31417929	rs72851123	intergenic	LINC01149(dist=3179),HCP5(dist=13028)	A	C	0.025	1.000	1.292	0.045	1.18E-08
PENICILLIN	6	31419604	rs72851131	intergenic	LINC01149(dist=4854),HCP5(dist=11353)	A	G	0.025	1.000	1.292	0.045	1.18E-08
PENICILLIN	6	31419619	rs72851189	intergenic	LINC01149(dist=4869),HCP5(dist=11338)	A	C	0.025	1.000	1.292	0.045	1.18E-08
PENICILLIN	6	31361005	rs139701319	intergenic	HLA-B(dist=36049),MICA-AS1(dist=1061)	G	A	0.025	0.997	1.292	0.045	1.22E-08
PENICILLIN	6	31407653	rs76151409	intergenic	MICA(dist=24561),LINC01149(dist=1791)	G	A	0.025	0.999	1.291	0.045	1.28E-08
PENICILLIN	6	31371342	rs2301750	intronic	MICA	C	G	0.025	0.999	1.291	0.045	1.32E-08
PENICILLIN	6	31327265	rs114783056	intergenic	HLA-B(dist=2309),MICA-AS1(dist=34801)	C	T	0.026	0.999	1.287	0.045	1.50E-08
PENICILLIN	6	31327326	rs68085422	intergenic	HLA-B(dist=2370),MICA-AS1(dist=34740)	G	A	0.026	0.999	1.287	0.045	1.50E-08
PENICILLIN	6	31327446	rs72502573	intergenic	HLA-B(dist=2490),MICA-AS1(dist=34620)	C	T	0.026	0.999	1.287	0.045	1.50E-08
PENICILLIN	6	31321657	rs3177747	UTR3	HLA-B(NM_005514:c.*421C>T)	G	A	0.026	0.965	1.284	0.044	1.62E-08
PENICILLIN	6	31326959	rs114166883	intergenic	HLA-B(dist=2003),MICA-AS1(dist=35107)	C	T	0.026	0.997	1.286	0.045	1.64E-08
PENICILLIN	6	31327114	rs72502572	intergenic	HLA-B(dist=2158),MICA-AS1(dist=34952)	T	C	0.026	0.997	1.286	0.045	1.64E-08
PENICILLIN	6	31321845	rs361531	UTR3	HLA-B(NM_005514:c.*233A>G)	T	C	0.025	0.997	1.287	0.045	1.79E-08
PENICILLIN	6	31413000	rs9765960	ncRNA_intronic	LINC01149	G	A	0.037	1.000	1.229	0.038	4.25E-08
SIMVASTATIN	3	145293340	rs76103438	intergenic	DIPK2A(dist=1582130),LNCSRLR(dist=490791)	T	A	0.025	0.899	1.870	0.112	2.56E-08
