# Supplementary material for: High-throughput framework for genetic analyses of adverse drug reactions using electronic health records
Source: PLoS Genet. 2021 Jun 1;17(6):e1009593. doi: 10.1371/journal.pgen.1009593 (PMC8195357; doi:10.1371/journal.pgen.1009593)
Supplement: S3 Table — (PDF) [file pgen.1009593.s003.pdf]

S3 Table. Trans-ethnic replication of lead variant per signal associated with adverse drug reactions in individuals with self-reported African ancestry.

Adverse Drug Reactions	Variant	Mapped Gene	Consequence	Allele [a]	EAF [b]	R^2	OR (95% CI) [c]	P
Aspirin	rs115346678	SSBP2, ATG10	intergenic	G/A	0.03	0.98	0.97 (0.52 to 1.41)	0.88
Cephalexin	rs34545984	LOC105376453, OTUD1	intergenic	G/T	< 0.005			
Codeine	rs9620007	WBP2NL	intronic	C/G	0.32	0.98	0.93 (0.81 to 1.06)	0.28
CYP2D6-metabolized opioids	rs62436463	OPRM1	intronic	C/T	0.02	0.94	0.77 (0.46 to 1.09)	0.11
	rs739296	SEPTIN3	intronic	G/A	0.30	0.99	0.97 (0.87 to 1.06)	0.45
Meperidine	rs11049274	PTHLH, LOC729291	intergenic	G/A	0.02	1.00	1.38 (0.53 to 2.23)	0.46
	rs113100019	FIP1L1	intronic	T/G	< 0.005			
	rs185462714	SERINC5	intronic	A/G	< 0.005			
Penicillin	rs115200108	HLA-B, MICA-AS1	intergenic	C/A	0.01	1.00	0.77 (0.29 to 1.25)	0.28
Simvastatin	rs76103438	DIPK2A, LNCSRLR	intergenic	T/A	0.01	0.90	1.89 (0.53 to 3.26)	0.36

EAF = Minor allele frequency; R^2 = imputation quality

[a] Alleles are listed as reference/effect and are reported in the forward strand.

[b] Variants with MAF < 0.005 were excluded from analysis.

[c] OR and 95% CIs were derived from logistic regression models adjusted for sex, age, length of electronic health records (years), and first 10 principal components.
