# Supplementary material for: High-throughput framework for genetic analyses of adverse drug reactions using electronic health records
Source: PLoS Genet. 2021 Jun 1;17(6):e1009593. doi: 10.1371/journal.pgen.1009593 (PMC8195357; doi:10.1371/journal.pgen.1009593)
Supplement: S4 Table — (PDF) [file pgen.1009593.s004.pdf]

S5 Table. Genome-wide significant variants associated with adverse drug reactions in self-reported African ancestry individuals.

ADVERSE DRUG REACTION	CHR	POS	SNP	FUNCTION	GENE	REF	ALT	EAF	R2	OR	SE	P
CYP2D6-Metabolized Opiod	1	63974562	rs146874207	intronic	ITGB3BP	T	A	0.0424181	0.98927	1.64392	0.0911337	4.91E-08
CYP2D6-Metabolized Opiod	1	63977576	rs138184320	intronic	ITGB3BP	A	T	0.0461987	0.99359	1.62984	0.0886776	3.62E-08
CYP2D6-Metabolized Opiod	1	64024507	rs143428350	intronic	EFCAB7	C	T	0.0431465	0.94358	1.66626	0.0903162	1.57E-08
CYP2D6-Metabolized Opiod	1	64042059	rs141490219	intergenic	EFCAB7(dist=3695),PGM1(dist=17023)	G	A	0.0424875	0.98206	1.64574	0.0911313	4.58E-08
CYP2D6-Metabolized Opiod	1	64066333	rs116486075	intronic	PGM1	C	T	0.0455397	0.94886	1.64483	0.0892045	2.42E-08
Erythromycin	19	53788036	rs189641101	downstream	FAM90A27P(dist=434)	A	T	0.0579564	0.91986	2.86106	0.183805	1.07E-08
Erythromycin	8	15422695	rs140359534	intronic	TUSC3	T	C	0.0612514	0.93249	2.80678	0.178575	7.50E-09
Simvastatin	7	91884628	rs12670516	intronic	ANKIB1	C	T	0.0856687	0.98559	2.81764	0.187978	3.57E-08
Simvastatin	7	91887996	rs1013522	intronic	ANKIB1	T	C	0.0856687	0.98559	2.81764	0.187978	3.57E-08
Simvastatin	7	91899508	rs4727271	intronic	ANKIB1	C	G	0.0852525	0.98384	2.82149	0.187959	3.42E-08
Simvastatin	7	91970821	rs76631110	intronic	ANKIB1	C	A	0.0863624	0.97071	2.83169	0.186898	2.56E-08
Simvastatin	9	25730652	rs1937930	intergenic	TUSC1(dist=51796),LINC01241(dist=49402)	T	G	0.0699917	0.86791	3.07349	0.198614	1.57E-08
Any Statin	14	105258893	rs2494749	intronic	AKT1	A	G	0.875555	0.77651	0.522439	0.115837	2.08E-08

OR and 95% CIs were derived from logistic regression models adjusted for sex, age, length of electronic health records (years), and first 10 principal components.
