# Supplementary material for: High-throughput framework for genetic analyses of adverse drug reactions using electronic health records
Source: PLoS Genet. 2021 Jun 1;17(6):e1009593. doi: 10.1371/journal.pgen.1009593 (PMC8195357; doi:10.1371/journal.pgen.1009593)
Supplement: S1 Fig — Red lines on Manhattan plots show the phenome-wide level of significance (5.0 × 10^−5^). Phenotypes with P-values < 0.005 were annotated. (PDF) [file pgen.1009593.s006.pdf]

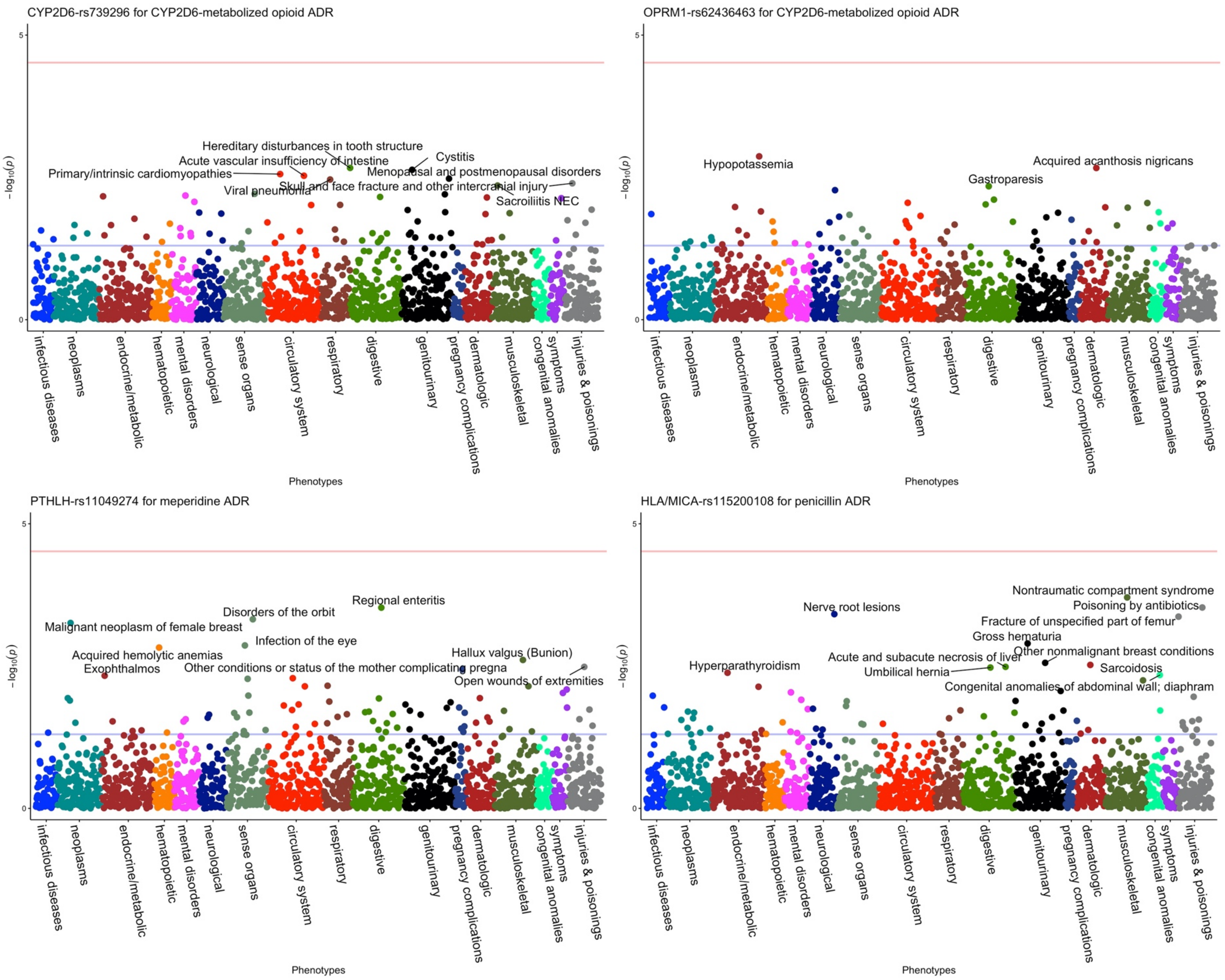

S1 Figure. Manhattan plots of phenome-wide analysis of lead variants in *CYP2D6*, *OPRM1*, *PTHLH*, and *HLA/MICA* associated with adverse drug reactions (ADRs). Red lines on Manhattan plots show the phenome-wide level of significance (5.0×10^{-5}). Phenotypes with P-values < 0.005 were annotated.
