# Supplementary material for: High-throughput framework for genetic analyses of adverse drug reactions using electronic health records
Source: PLoS Genet. 2021 Jun 1;17(6):e1009593. doi: 10.1371/journal.pgen.1009593 (PMC8195357; doi:10.1371/journal.pgen.1009593)
Supplement: Attachment — Submitted filename: ADR_response.docx [file pgen.1009593.s007.docx]

April 14, 2021

Gregory S. Barsh, PhD

Gregory P. Copenhaver, PhD

Editors-in-Chief

*PLOS Genetics*

**Re: PGENETICS-D-20-01665 High-throughput genetic analyses of adverse drug reactions using electronic health records**

Dear Dr. Barsh & Dr. Copenhaver:

We thank the reviewers for their insightful comments regarding our manuscript. We have revised the manuscript to address each of their comments. Our modifications are listed below and are highlighted in yellow in the revised manuscript.

**Reviewer: 1**

**While I agree that the drug allergy section in the EHR often lists ADRs, true allergies are also reported and captured (PMID:30100688 Figure 2). I am concerned that combining anything listed in this section for a certain drug does not provide an appropriate phenotype. Given that allergy data is not uniformly collected and/or reported and can represent everything from mild, expected, patient-reported side effects to hypersensitivity reactions, knowing the types of “allergies” reported is important to be able to determine risk-benefit. Grouping these effects in this manner assumes that genetic factors associated with gastrointestinal patient-reported side effects from antibiotics are the same that contribute to the development of Stevens Johnson Syndrome or other hypersensitivity reactions.**

Thank you. We agree that the data in the allergy sections of the EHRs often could combine different phenotypes. These points have been acknowledged as a limitation in the **Discussion**. However, the genetic signals identified in our analyses are dominated by the more frequent phenotypes (e.g., rash from penicillin, GI distress from codeine) since rare and known *HLA*-restricted phenotypes, such as Stevens-Johnson syndrome, that occur at an incidence of 1-5/million per year are unlikely to create a significant signal. We have clarified this point in paragraph 8 of the **Discussion** (pg. 15).

**While the authors address the inability to ascertain the specific drug reaction as a limitation, they also state that in their previous study (Ariosto 2014), the majority of patients had gastrointestinal effects when opioid allergy alerts were evaluated. Therefore, it seems that the type of allergy/ADR can be obtained in some circumstances. Would like to see a breakdown of the types of drug reactions that were included for each drug/drug class when this data was available. Without this information, it is difficult to determine (1) if the phenotype was appropriate for the GWAS and (2) what the results actually indicate.**

Thank you. As suggested, we have now included a supplementary table (renumbered **S1 Table**), which breaks down the types of drug reactions for each of the drug/drug classes. As shown the in the table, there is a significant proportion of patients with missing reaction type information, ranging from 24.4% to 58.0%. Genetic analyses for specific types of drug reactions yielded significant results only for nausea or vomiting related to CYP2D6-metabolized opioids, which was the same signal identified for any CYP2D6-metabolized opioid reaction. This is consistent with similar findings in Mountain et al. 2013 (reference 25) in a 23andMe cohort. Corresponding text has been included in paragraph 2 of the **Results** (pg. 5) and paragraph 3 of the **Discussion** (pg. 12-13).

Therefore, our framework is capable of identifying genetic association signals for specific types of adverse drug reactions, but the current quality of reaction information severely limits the sample size for high-throughput analyses. As discussed in paragraph 9 of the **Discussion** (pg. 15), emphasizing the importance of detailing the type of reaction and improving current EHR systems to standardize inputs will enable our framework to perform more of these sorts of genetic analyses in the future.

**Broadly indicating a genetic variant is associated with an ADR does not provide any information on the potential risk of the medication for the patient. Expected GI side effects and hypersensitivity reactions would not result in the same clinical management decisions and deeming a patient "at risk" for an ADR/allergy could cause the patient to be given suboptimal therapy when the risk was actually only a common, expected side effect. Can the authors comment on this, as this was not addressed in the manuscript, and is a major limitation.**

We agree that further follow-up on the genetic variants identified by our high-throughput framework is needed to fully understand the risks of ADRs in patients. However, we believe that this can provide a roadmap of an approach and that future improvements in allergy documenting in EHRs will enable analyses of more specific ADR phenotypes with our framework. We have discussed this limitation in paragraph 8 of the **Discussion** (pg. 15).

**Page 12: The authors state that “utilizing EHRs allowed us to study ADRs in individuals with diverse clinical and ethnic backgrounds.” However, the GWAS was not conducted in patients of African ancestry and the identified associations were not validated in these patients, so this did not seem to be the best the method to study ADRs in non-Europeans. I am wondering (1) If the top 10 most reported drugs in the allergy section were the same for patients of European and African ancestry, and (2) given that there are known ancestral differences in susceptibility to certain ADRs (e.g., ACE inhibitor-induced angioedema in African ancestry), other than sample size, was there a reason the GWAS not also conducted in the African ancestry individuals?**

The top 10 most reported drugs in the allergy section were the same for patients of European and African ancestry with some minor differences in ordering, as noted now in paragraph 2 of the **Results** (pg. 5).

We excluded GWAS with the African ancestry cohort due to limited sample size, which can lead to unstable point estimates and inflated false discovery rates. Therefore, further work is needed to determine the generalizability of our findings in European ancestry individuals. Due to the high-throughput nature of our framework, it should be easy to apply to other large multi-ancestry EHR-based biobanks for future genetic analyses. Nonetheless, we agree that the GWAS results from African ancestry individuals may be informative and have therefore included the significant variants in **S4 Table**. Corresponding text has been added to paragraph 1 of the *Genome-wide analysis* subsection of the **Results** (pg. 6). We have also emphasized the small sample size and European-ancestry-dominated reference panel as a limitation in paragraph 6 of the **Discussion** (pg. 14).

**Given that the associations did not validate in African Americans, was any attempt made to validate the identified associations in individuals of European ancestry? Since previously identified and replicated associations for statin-induced myopathy (i.e., SLCO1B1) and aspirin-associated ADRs, such as aspirin-induced asthma (HLA-DPB1), were not identified in the study, I am wondering if this could be due to how the drug allergy phenotype was defined, or if the authors have another explanation.**

We contributed data from BioVU to the meta-analysis in the Krebs et al. 2020 (reference 29), which validated our phenotype and results in cohorts from the UK Biobank, the Estonian biobank, and 23andMe. The CYP2D6-metabolized opioid ADR results were also previously validated by Mountain et al. 2013 (reference 25) in a 23andMe cohort.

We acknowledge that the quality of data regarding type of adverse reaction is currently limited. For instance, there were only 55 cases of aspirin-induced asthma in our cohort. The previous linkage of a SNP in the *HLA-DPB1* region with aspirin exacerbated respiratory disease was also in a Korean population and not necessarily generalizable to Europeans (PMID: 23180272). The *SLCO1B1*5* variant (rs4149056) was significantly associated with increased risk of simvastatin ADR (OR = 1.12, P = 0.047), but did not reach genome-wide significance. There is strong evidence for the association between *SLCO1B1*5* and simvastatin-induced myopathy (particularly high doses i.e., 80 mg), but it is less clear for other statins (PMID: 22617227). Atorvastatin is more commonly prescribed in BioVU than simvastatin (see **Table 2**) and there are relatively few patients taking 80 mg simvastatin doses (PMID: 24096969), which may have decreased the power of our analysis. Additionally, there our framework is currently unable to relate dose to incidence of ADR, which we acknowledge as a limitation in paragraph 8 of the **Discussion** (pg. 15).

**Reviewer: 2**

Major comments

**While a strength of the paper is the considerable size, the reported findings are confirmatory and lack novelty unfortunately. In fact, the paper is more a method validation than a discovery study and this should be reflected in the title and throughout the paper. Therefore, I would encourage to show more data on the text mining of the “allergy section”**

As suggested, we have changed the title and abstract to emphasize the methodology presented by the paper. We have included more information on the types of adverse drug reactions retrieved from the “allergy section” of the electronic health records in **S1 Table**. We have additionally included **S5 Table**, which includes all the regular expressions used to extract the adverse drug reactions.

**It is not clear to me how the 14 drugs/groups were selected. This should be described in more detail. From the title I would have thought that the authors for instance would have focused on severe hypersensitivity reactions i.e., Stevens–Johnson syndrome, toxic epidermal necrolysis, drug-induced liver injury.**

We first included the top 10 most frequently appearing drugs in the “allergy section.” We additionally included ADRs for any statin, atorvastatin, and simvastatin since they appeared frequently and share a similar metabolic pathway. Likewise, we included CYP2D6-metabolized opioid prodrugs as a grouped analysis due to a shared metabolic pathway. Description of the reasoning behind including the 14 drugs or drug groups was included in paragraph 2 of the **Results** (pg. 5).

Our framework does not currently have enough power to pick up rarer *HLA*-restricted phenotypes such as Stevens-Johnson syndrome or drug-induced liver injury which occur at an incidence of <5/million per year. However, it has been validated in more common phenotypes such as penicillin reactions and CYP2D6-metabolized opioid reactions as discussed above.

**In the introduction the authors correctly address the challenge that “Drug response phenotypes, such as ADRs, are less often recorded than physiological traits and common diseases” To be able to assess drug response phenotypes detailed information regarding dose, interval and time relation with occurrence of the ADR is important. Was more detailed information regarding dose and time of administration available from the EHR and considered for inclusion in the analysis?**

Drug allergy labels in the “allergy section” of the clinical notes are often self-reported and recorded by the healthcare provider. Therefore, the information is not linked to a specific dose or time of administration. We have acknowledged this limitation in paragraph 8 of **Discussion** (pg. 15).

**The authors correct for multiple testing of the genetic variants, but it is not clear how many endpoints were tested and if this is corrected for.**

We tested a total of 14 ADR endpoints, including one for each of the 14 selected drug or drug groups. We did not apply a correction to the P-values and used *P* < 5 × 10^-8^ as our threshold for genome-wide significance.

**The methods section is quite brief and should be expanded. For example, it would be useful to include detailed information on the identification of ADRs from the allergy sections free text.**

As mentioned above, we have now included all regular expressions used to extract the ADRs in **S5 Table**. We have also added additional details on the identification of ADRS to paragraphs 1-2 of the **Methods** (pg. 16).

Minor comments

**The paper is very brief and little detail is provided. For example, the results section on the PheWAS is only 2 sentences.**

The PheWAS was included to demonstrate that our ADR phenotypes can be more useful than diagnosis codes, which may have limited power or simply do not cover the ADR of interest. We have clarified this point in the *Phenome-wide analyses* subsection of the **Results** (pg. 11) and paragraph 4 of the **Discussion** (pg. 13).

**The figures are of low resolution and should be improved**

Lower resolution figures were included in the initial submission to accommodate for file size restrictions. The final version of the figures have higher resolution.

Thank you for taking the time to consider our paper.

Sincerely,

Neil S. Zheng, BS	Trainee in Biomedical Informatics

Wei-Qi Wei, MD, PhD, FAMIA	Assistant Professor of Biomedical Informatics

Department of Biomedical Informatics,

Vanderbilt University Medical Center,

2525 West End Avenue Suite 1500,

Nashville, TN 37232-6602, USA;

Phone: (615) 343-1956;

E-mail: [wei-qi.wei@vumc.org](mailto:wei-qi.wei@vumc.org)
